# Characterization of the latent membrane protein 1 signaling complex of Epstein-Barr virus in the membrane of mammalian cells with bimolecular fluorescence complementation

**DOI:** 10.1186/1743-422X-8-414

**Published:** 2011-08-24

**Authors:** Pooja Talaty, Amanda Emery, David N Everly

**Affiliations:** 1 Department of Microbiology and Immunology, Chicago Medical School, Rosalind Franklin University of Medicine and Science, 3333 Green Bay Road, North Chicago, Illinois, 60064, USA

**Keywords:** Epstein-Barr Virus, latent membrane protein 1, bimolecular fluorescence complementation, TRAF2, TRAF3

## Abstract

**Background:**

Bimolecular fluorescence complementation (BiFC) is a novel technique to examine protein-protein interaction through the assembly of fluorescent proteins. In the present study, BiFC was used to study the assembly of the Epstein-Barr virus latent membrane protein 1 (LMP1) signaling complex within the membrane of mammalian cells. LMP1 signaling requires oligomerization, localization to lipid rafts, and association of the cytoplasmic domain to adaptor proteins, such as the tumor necrosis factor receptor associated factors (TRAFs).

**Methods:**

LMP1-TRAF and LMP1-LMP1 interactions were assayed by BiFC using fluorescence microscopy and flow cytometry. Function of LMP1 BiFC contructs were confirmed by transformation assays and nuclear factor- κB (NF-κB) reporter assays.

**Results:**

BiFC was observed between LMP1 and TRAF2 or TRAF3 and mutation of the LMP1 signaling domains reduced complementation. Fluorescence was observed in previously described LMP1 signaling locations. Oligomerization of LMP1 with itself induced complementation and BiFC. LMP1-BiFC constructs were fully functional in rodent fibroblast transformation assays and activation of NF-κB reporter activity. The BiFC domain partially suppressed some LMP1 mutant phenotypes.

**Conclusions:**

Together these data suggest that BiFC is a unique and novel platform to identify and characterize proteins recruited to the LMP1-signaling complex.

## Background

Epstein-Barr virus (EBV) is a DNA tumor virus that latently infects and immortalizes B-lymphocytes. The latent membrane proteins of EBV induce constitutive signaling to establish latency and ensure the survival of the infected cell [1,2]. Latent membrane protein 1 (LMP1) of EBV is termed the EBV oncogene as it is required for EBV B-cell transformation and sufficient to transform rodent fibroblasts [3-9]. LMP1 expression is also frequently detected in the cancers associated with EBV [1,2,10-12]. It alters the cellular environment by inducing a number of signaling pathways, including nuclear factor- κ B (NF-κB), phosphoinositide 3-kinase (PI3K), mitogen-activated protein kinase, and c-Jun N-terminal kinase [6,7,13-19].

LMP1 has a short cytoplasmic amino terminus, a six pass transmembrane domain, and a cytoplasmic carboxyl-terminal signaling domain. The transmembrane domain is required for ligand-independent self-association and localization to lipid raft domains of the membrane [20-26]. Mutations in the membrane domain that impair LMP1 raft localization can block signaling [22,27-29]. LMP1 signaling is initiated by binding of adaptor proteins to the two carboxyl-terminal activating regions (CTARs), CTAR1 and CTAR2. CTAR1 binds tumor necrosis factor receptor-associated factors (TRAF)1, TRAF2, TRAF3, and TRAF5 [30]. CTAR2 binds other adaptors, including TNFR-associated death domain and receptor interacting protein 1, that in turn recruit TRAF2 and TRAF6 [15,31]. Interferon regulatory factor 7 is also recruited to CTAR2 and is activated by TRAF6-dependent ubiquitylation [32-34]. Although it is clear that LMP1 signaling requires the TRAFs and other adaptor proteins, downstream proteins recruited to the LMP1 signaling complex continue to be defined.

The CTAR1 domain is critical for activation of discrete signaling pathways and cellular phenotypes. Activation of PI3K and extracellular-signal-regulated kinase (ERK) signaling through CTAR1 correlates with fibroblast transformation and epithelial cell motility and invasion [5-7,35-37]. Inhibition of PI3K or ERK signaling blocks these effects. Activation of PI3K signaling is correlated with regulation of several proteins important for promoting cell cycle progression [5-7]. LMP1 downregulates p27KIP1 transcriptionally through the effects of a repressive E2F complex, E2F4/p130 [38]. Several other pathways have recently been associated with CTAR1 ERK activation, including STAT3, PKC(delta), and non-canonical NF-κB (p50/p50/Bcl3 complexes) [39-42]. The exact mechanisms of LMP1-induced signaling through CTAR1 to induce transformation and cell cycle have not been fully elucidated.

Understanding the dynamic molecular events within the membrane resulting in LMP1 signaling is a complex and difficult biological problem. In recent years a number of enzyme and fluorescence based complementation assay have been developed that can be applied to membrane proteins [43]. In bimolecular fluorescence complementation (BiFC) interacting proteins are expressed as fusion proteins with fragments of yellow fluorescence protein (YFP) [44]. Individually proteins do not possess intrinsic fluorescence but interaction between proteins leads to assembly of functional YFP which can be detected by fluorescence based techniques, such as fluorescence microscopy and flow cytometry.

The goal of the current study was to examine the assembly of the LMP1 signaling complex using BiFC. Fluorescence complementation was observed for LMP1 with TRAF2, LMP1 with TRAF3, and LMP1 with itself. Fluorescence was localized to perinuclear and membrane regions of the cell which is consistent with previously described localization of LMP1 signaling complexes. Mutations in CTAR1 and CTAR2 decreased the complementation of LMP1 with the TRAFs. LMP1 containing the YFP domain was fully functional in rodent fibroblast transformation and in the induction of NF-κB reporter plasmids. These results suggest that BiFC is an attractive way to analyze the assembly of signaling complexes with full-length LMP1 protein and to understand the contribution of the membrane portion of LMP1 to signaling.

## Methods

### Plasmids

BiFC plasmids encoding Venus YFP fusion proteins were constructed by Stephen W. Michnick (University of Montreal, Montreal, Quebec, Canada [45]). The plasmids contain the leucine zipper domain (zip) of the yeast protein, general control nondepressible 4, fused at the amino or carboxyl termini to the amino (NYFP) or carboxyl (CYFP) fragment of Venus YFP. The zip domain is fused at either end of each YFP fragment, zip-NYFP, zip-CYFP, NYFP-zip, and CYFP-zip. The zip and YFP coding sequences are flanked by unique restriction enzyme sites and separated by a sequence encoding a 10 amino acid linker (GGGGSGGGGS). LMP1 and TRAF sequences were cloned by PCR using primers containing the appropriate restriction enzymes to replace the zip domain and maintain proper coding frame with the YFP sequences. All constructs described below were completely sequenced to confirm the desired clones.

LMP1-binding TRAF2 and TRAF3 constructs have been previously described [7]. TRAF2 (amino acids 98-501, NM_021138) and TRAF3 (amino acids 345-568, NM_003300) contain the LMP1-binding TRAF domain but lack RING (really interesting new gene) and zinc finger domains. Constructs with CYFP at the C-terminus of the myc-tagged TRAFs were cloned with CYFP to create TRAF2-CYFP and TRAF3-CYFP. Fusions at the N-terminus of the TRAFs were cloned to create CYFP-TRAF2 and CYFP-TRAF3. N-terminally tagged TRAFs lack the myc-epitope tag.

Full-length LMP1 from B95-8 strain EBV (K02165) was cloned with NYFP and CYFP in various orientations. Previously characterized LMP1 mutants impaired in LMP1 signaling were also cloned into BiFC vectors [5-7]. Mutants include LMP1-A5 (A5) which contains CTAR1 mutations (amino acids 204-208 PQQAT mutated to AAAAA). LMP1-Y384G (Y384G) contains a CTAR2 point mutation (Y 384 to G) that abrogates CTAR2 signaling [46,47]. A5-Y384G contains point mutations in both CTAR1 and CTAR2. 1-231 containing amino acids 1 through 231 is deleted for CTAR2 but contains CTAR1, while 1-231-A5 is deleted for CTAR2 and contains the A5 mutations in CTAR1. The 1-187 mutant is deleted for both CTAR1 and CTAR2 and contains only the transmembrane domain. The mutant CTAR1/2 contains only the cytoplasmic signaling domain of LMP1 from amino acids 184 to 386 and lacks the membrane domain. The expression vector pmCherry-N1 encoding the red fluorescent protein, mCherry, was purchased from Clontech. The BiFC expression cassette encoding LMP1-NYFP was subcloned into retrovirus packaging vector pBabe-puro for use in transformation assays.

### Cell Culture, Transfections, and Retrovirus

Human embryonic kidney-293T (HEK-293T) and Rat-1, rodent fibroblasts, cells were maintained in Dulbecco modified Eagle medium (Mediatech) supplemented with antibiotic/antimycotic mixture and 10% (v/v) heat-inactivated fetal bovine serum. Cells were transfected with Transit-LT1 (Mirus) according to the manufacturer's directions. Retrovirus production was accomplished as previously described [38] by transfection of HEK-293T cells with control (Babe-puro), LMP1, or LMP1-NYFP packaging vectors with plasmids expressing vesicular stomatitis virus G protein and gag-pol from Moloney murine leukemia virus. Twenty-four hours post-transfection media was changed and cells were moved to 33°C. Fourty-eight hours post-transfection clarified supernatents were collected and used to infect Rat-1 cells. Retrovirus infection was performed in the presence of 8 μg/ml polybrene. Stably transduced Rat-1 cells were selected with puromycin (5 μg/ml, Mediatech).

### Western blotting

Cells were washed with ice cold phosphate buffered saline (PBS, Mediatech) and lysed with radio immunoprecipitation assay buffer (10 mM Tris-HCl, pH 8.0, 140 mM NaCl, 1% Triton X-100, 0.1% sodium dodecyl sulfate (SDS), 1% deoxycholic acid, protease and phosphatase inhibitors (Pierce)). Cell lysates were clarified by centrifugation and quantitated by Bio-Rad DC protein assay system (Bio-Rad). Samples were then boiled in SDS sample buffer and indicated amounts of protein were separated using SDS-polyacrylamide gel electrophoresis, and transferred to nitrocellulose membranes (LiCor) for western blotting analysis. LMP1 was detected with a mixture of four rat monoclonal antibodies diluted 1:500 each (Cao 7E10, Cao 8G3, LMP1 IG6, and Cao 7G8, Ascenion GmbH). TRAF2 and TRAF3 antibodies were purchased from Santa Cruz. Fusion proteins were detected with myc-tag antibody (Upstate) and YFP antibodies (632460 polyclonal for NYFP and CYFP and 632381 monoclonal for CYFP from Clontech). Primary antibodies were detected with IRDye™ labeled secondary antibodies (Li-Cor) and scanning with a Li-Cor Odyssey imaging system. Bands were quantitated using the Li-Cor imaging software.

### BiFC Assays

Different combinations of BiFC plasmids were transfected into HEK-293T cells and examined by fluorescence techniques. For fluorescent microscopy, cells were plated on coverslips and fixed 24 hours post-transfection with 4% paraformaldehyde in PBS for ten minutes at room temperature and washed with PBS. Coverslips were mounted with ProLong Gold Antifade reagent containing 4'-6-Diamidino-2-phenylindole (DAPI, Invitrogen). Cells were examined at low magnification for YFP fluorescence. High resolution images were acquired using the Olympus Fluoview 300 confocal microscope at the microscopy core of Rosalind Franklin University of Medicine and Science (RFUMS) at 60 × objective under oil immersion. Analysis was performed using Fluoview software (Olympus, Melville, NY).

Cells used for flow cytometry were co-transfected with pmCherry-N1 (Clontech) to enrich for transfected cells. Forty-eight hours post-tranfection cells were trypsinized, washed, and resuspended in PBS. Fluorescence was determined using the LSRII Flow Cytometer (BD Biosciences) in the Flow Cytometry Core Facilty of RFUMS. The main cell population was gated using the forward scatter versus side scatter dot plot. Transfected cells were enriched by gating for mCherry fluorescent cells. YFP gating was determined by comparing the histograms of mCherry alone transfected cells with BiFC plasmid transfected cells. 1 × 10^4 ^mCherry positive cells were analyzed for each combination of plasmids and the mean fluorescent intensity (MFI) of YFP was determined. Flow cytometry data was analyzed with BD FACSDiva (BD Biosciences) and FlowJo (Tree Star) software. Cells were also harvested for western blotting to confirm expression of BiFC plasmids.

### Reporter Assays

Reporter assays were performed as previously described [38]. HEK-293T cells were plated 1:5 into 12-well plates one day prior to transfection. Cells were transfected with 0.2 μg of pRL-SV40 (Renilla luciferase) (Promega), 0.2 μg of pNF-κB-Luc (Stratagene), and 0.2 μg of vector or LMP1-expressing plasmids. Forty hours post-transfection cells were harvested and luciferase activity was assayed using the Dual-Luciferase^® ^Reporter Assay System (Promega) according to the manufacturer's directions. Relative luciferase activity was determined by dividing the firefly luciferase activity of the NF-κB promoter constructs by the internal control *Renilla *luciferase activity. Each condition was done in triplicate and replicated in at least three experiments.

### Transformation Assays

Transformation assays were performed as previously described [6]. Focus formation assays were performed by infection of subconfluent monolayers of Rat-1 cells followed by growth for 10 days to allow for focus formation. Foci were fixed and stained with 1% (w/v) crystal violet in 50% (v/v) ethanol. For colony formation assays stable Rat-1 cells were seeded in soft agar in triplicate at 5 × 10^5^/well. Colonies were grown for 10 days and stained with MTT (methylthiazolyldiphenyl-tetrazolium bromide, Sigma) for one hour. Subconfluent stable cells were stained with crystal violet as above and observed for transformed phenotype. Cellular phenotypes were observed and documented using a Leica EZ4D dissecting microscope with integrated digital camera.

## Results

### BiFC with the LMP1 cytoplasmic domain and TRAFs

Binding between LMP1 and the TRAFs was previously identified using the cytoplasmic domain of LMP1 in yeast two-hybrid (Y2H) screens [48]. To determine if LMP1 + TRAF2 or TRAF3 binding induces fluorescence complementation, BiFC assays were performed. LMP1, TRAF2, and TRAF3 were cloned into BiFC expression plasmids as fusion proteins with the amino-terminus of YFP (NYFP) or the carboxyl-terminus of YFP (CYFP). Constructs are named for the protein and YFP domain that they contain in the order in which they are encoded. NYFP-CTAR1/2 contains the amino-terminus of YFP fused to the cytoplasmic domain of LMP1 (amino acids 184-386, containing CTAR1 and CTAR2). TRAF2 and TRAF3 fusion proteins with CYFP at the amino-termini, CYFP-TRAF2 and CYFP-TRAF3, were tested. TRAFs contain several conserved domains, including Zn-RING, Zn-fingers, TRAF-N and TRAF-C domains. The TRAF-N and TRAF-C domains bind the signaling domains of LMP1 and other proteins. The zinc-binding domains also mediate protein-protein interaction and can function as E3 ubiquitin ligases. Since the TRAFs function as E3-ubiquitin ligases that induce signaling and sometimes turnover [49,50], previously described truncated TRAFs that lack the E3-ubiquitin ligase domain but maintain the TRAF-N and TRAF-C LMP1-binding domains were used [7]. Although these TRAFs function as dominant negatives in the activation of downstream signaling, they maintain LMP1-binding but avoid possible complications in subsequent experiments related to their ubiquitin ligase activity.

**Figure 1:**
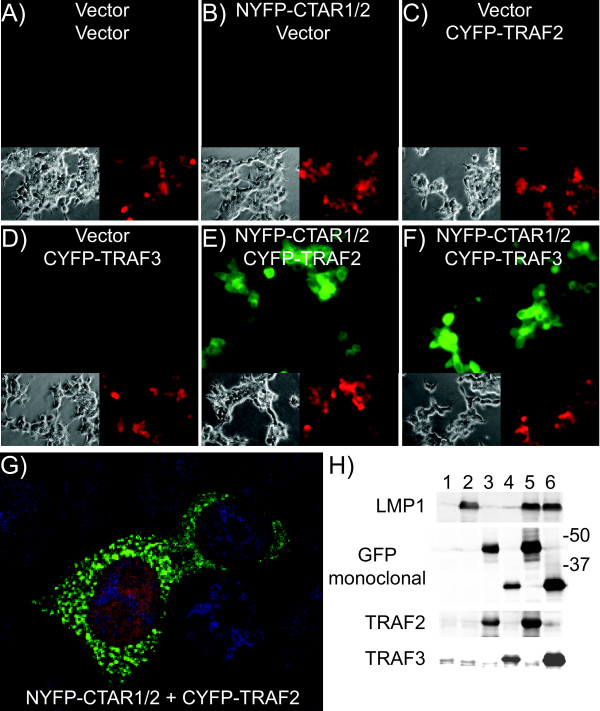
**BiFC with LMP1 cytoplasmic domain and TRAFs**. HEK-293T cells were transfected with the indicated LMP1 + TRAF pairs and transfection control, pmCherry. NYFP-CTAR1/2 expresses the N-terminus of YFP (NYFP) fused to the cytoplasmic domain of LMP1, amino acids 184-386, at the N-terminus. CYFP-TRAF2 and CYFP-TRAF3 express the CYFP fused to TRAF2 and TRAF3, respectively, at their N-termini. Twenty-four hours post-transfection cells were fixed and examined by fluorescence microscopy for transfection (mCherry expression) in the red panels and fluorescence complementation in the green panels (panels A, B, C, D, E, and F). Representative phase contrast and fluorescence micrographs are shown. High resolution image of NYFP-CTAR1/2 + CYFP-TRAF2 cells were counter stained with DAPI and acquired using a confocal microscope (panel G). In parallel, cells were assayed fourty-eight hours post-transfection by western blotting for protein expression. Lysates were blotted with GFP monoclonal (recognizes the CYFP domain), LMP1, TRAF2, and TRAF3 antibodies (panel H). Lanes 1-6 correspond to the combinations in panels A-F, respectively. Locations of molecular weight markers are indicated. Fifty micrograms of protein were loaded in each lane.

BiFC was determined between NYFP-CTAR1/2 and TRAF fusion proteins by co-transfection into HEK-293T cells and fluorescence microscopy individually or in combination (Figure 1). Fluorescence was not observed in cells transfected with individual plasmids in combination with empty vector plasmid (Figure 1A, B, C, and D). Combinations of the LMP1 cytoplasmic domain with the TRAFs induced strong fluorescence (Figure 1E and 1F). The fluorescence was punctuate throughout the cytoplasm and excluded from the nuclei (Figure 1G). Similar results were obtained with TRAFs tagged at their carboxyl-termini (data not shown).

In parallel, transfected cells were harvested for western blotting (Figure 1H). Blotting with LMP1 specific antibody confirmed expression of NYFP-CTAR1/2 at about 50 kilodaltons (kDa) in lanes 2, 5, and 6. Strong bands at 50 and 30 kDa bound with a monoclonal GFP antibody, which only reacts with CYFP. Bands of 50 and 30 kDa are consistent with predicted size of CYFP-TRAF2 (lane 3 and 5) and CYFP-TRAF3 (lane 4 and 6), respectively. Much fainter bands were also observed in LMP1 and GFP blots at the appropriate molecular weights for the transfected constructs. These bands are likely the result of a small amount of spillover between lanes and strong reactivity of LMP1 and GFP antibodies. Blotting with TRAF2 and TRAF3 antibodies confirmed the identity of the TRAF2 and TRAF3 fusion proteins (Figure 1H, lanes 3 and 5 (TRAF2), and 4 and 6 (TRAF3)). These data demonstrate BiFC between the cytoplasmic domain of LMP1 with TRAF2 and TRAF3 tagged with NYFP and CYFP, respectively, and that the complementation occurred regardless of position of the CYFP domain relative to the TRAFs.

### BiFC between full-length LMP1 and the TRAFs

**Figure 2:**
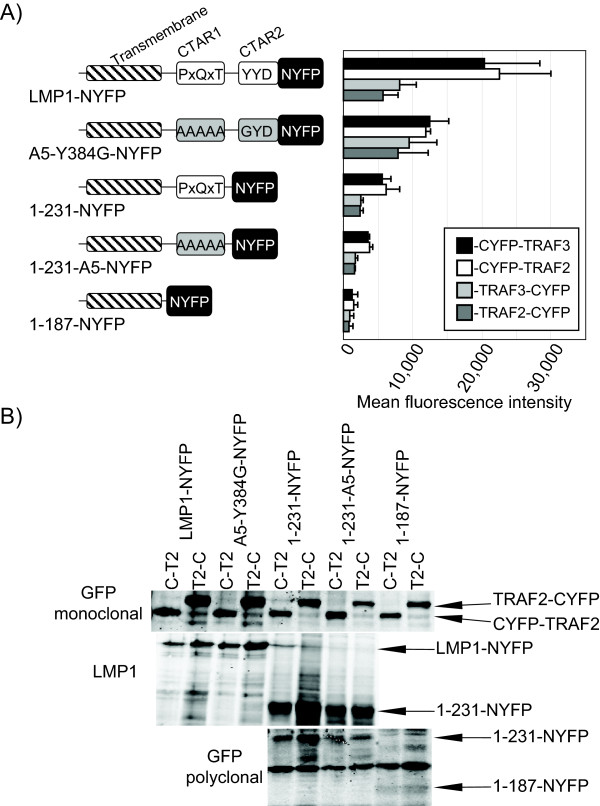
**BiFC between full-length LMP1 and the TRAFs**. HEK-293T cells were transfected with the indicated LMP1 + TRAF pairs. LMP1-NYFP expresses LMP1 fused to NYFP at the C-terminus of LMP1. The structure of LMP1 mutant and truncated constructs are indicated, including transmembrane domain (hatched box), CTAR1 (PxQxT), CTAR2 (YYD), mutant CTAR1 (gray AAAAA), mutant CTAR2 (gray GYD), and NYFP. A5-Y384G is a full-length mutant containing mutations in CTAR1 and CTAR2, 1-231 and 1-231-A5 are truncation mutants that are deleted for CTAR2 with wild-type or mutant CTAR1, respectively, and 1-187 expresses only the transmembrane domain of LMP1 fused to NYFP. CYFP-TRAF2 and CYFP-TRAF3 express the CYFP at the amino-terminus of the TRAFs and TRAF2-CYFP and TRAF3-CYFP express CYFP at the carboxyl-terminus of the TRAFs. Fluorescence was quantitated by flow cytometry. BiFC constructs were co-transfected with a mCherry expressing plasmid and after 48 hours, cells were trypsinized, washed, and resuspended in PBS. Cells were analyzed for mCherry and YFP fluorescence by flow cytometry. The main cell population was gated using the forward scatter versus side scatter dot plot and transfected cells were enriched by gating for mCherry fluorescent cells. For each combination, the mean fluorescent intensity (MFI) of YFP fluorescence for 1 × 10^4 ^mCherry positive cells was determined (panel A). MFI was determined in at least three independent experiments and error bars represent the standard deviation from the mean. In parallel protein expression was confirmed by western blotting for LMP1 and TRAF2 constructs (panel B). TRAF2-CYFP (T2-C) and CYFP-TRAF2 (C-T2) are recognized by a GFP monoclonal that binds CYFP. TRAF2-CYFP contains a tandem triple-myc tag that increases its molecular weight. LMP1 constructs were recognized with LMP1 and GFP polyclonal antibody that recognizes the NYFP domain.

Compared to traditional Y2H which requires nuclear localization, BiFC does not require nuclear localization and can be applied to membrane proteins [43]. Full-length LMP1 and TRAF2 and TRAF3 in various combinations were tested for BiFC (Figure 2). Because different combinations and configurations of fusion proteins may be required to obtain BiFC, cells were transfected with TRAFs tagged at the amino-terminus or carboxyl-terminus with the CYFP domain. To quantitate the relative fluorescence intensity of the different BiFC combinations flow cytometry was performed. Cells were transfected with the BiFC plasmids along with a plasmid expressing the mCherry protein (Figure 2A). Cells were harvested and transfected cells were analyzed by flow cytometry by gating the main cell population followed by cells with red fluorescence, i.e. mCherry positive cells. YFP fluorescence intensity was determined for 1 × 10^4 ^mCherry positive cells. The MFI of at least three replicates for each combination were averaged and plotted in Figure 2A.

Fluorescence levels were generally correlated with the whether LMP1 or TRAFs were tagged at their carboxyl- or amino-termini with the YFP domains. Brighter fluorescence was observed with the TRAFs tagged at the amino-terminus with CYFP, LMP1-NYFP + CYFP-TRAF2 and CYFP-TRAF3 (Figure 2A, black and white bars, respectively), compared to LMP1-NYFP + TRAF2-CYFP and TRAF3-CYFP (Figure 2A, gray bars). LMP1 tagged at the carboxyl-terminus with NYFP (LMP1-NYFP) had more than 10-fold greater fluorescence than LMP1 fusion proteins with the YFP domain at amino-terminus of LMP1 (data not shown). LMP1-NYFP + CYFP-TRAF2 or CYFP-TRAF3 are the combinations that induced the greatest fluorescence.

Decreased fluorescence complementation could be the result of steric interference with YFP domain association or could be due to differences in the expression of the different constructs. Expression levels of BiFC proteins were determined by western blotting for BiFC proteins (Figure 2B). Expression of fusion proteins was not correlated with their fluorescence. LMP1-NYFP expression was slightly increased in combination with TRAF2-CYFP compared to CYFP-TRAF2 (Figure 2B, LMP1-NYFP T2-C and C-T2, respectively). Similarly, expression of TRAF2-CYFP was slightly greater than CYFP-TRAF2. Similar expression patterns were observed for TRAF3 combinations (data not shown). Because expression of BiFC constructs was not correlated to their fluorescence, this suggests that the differences in BiFC are a result of steric interference with BiFC assembly rather than a result of the construct expression.

### Decreased BiFC with LMP1 Signaling Mutants

LMP1 point mutants were tested for fluorescence complementation with TRAF2 and TRAF3 and quantitated using flow cytometry (Figure 2A). Full-length mutant A5-Y384G contains alanine substitutions in the PxQxT motif of CTAR1 and a tyrosine to glycine substitution in CTAR2. LMP1-NYFP and A5-Y384G-NYFP were tested with TRAF2 and TRAF3 tagged at the amino-terminus with CYFP (Figure 2A, white and black bars, respectively). TRAF2 is known to bind both CTAR1 and CTAR2 during LMP1-mediated signaling. Fluorescence with CYFP-TRAF2 (Figure 2A, white bars) was reduced by mutation of both CTAR1 and CTAR2 (A5-Y384G). TRAF3 only binds to CTAR1 but not CTAR2. CYFP-TRAF3 fluorescence was also reduced by A5-Y384G (Figure 2A, black bars). Western blotting for LMP1 and TRAF2 constructs indicates that LMP1 and A5-Y384G and CYFP-TRAF2 constructs were equally expressed and that the decrease in fluorescence was not due to lower protein expression (Figure 2B). Similar expression levels were observed for TRAF3 constructs (data not shown). Although there was about a 50% decrease in fluorescence with A5-Y384G compared to wild-type LMP1, it was surprising that mutation of CTAR1 and CTAR2 did not completely abolish BiFC with LMP1 and TRAFs.

In order to further characterize BiFC between LMP1 and the TRAFs, LMP1 deletions that lack CTAR2 but containing CTAR1 (1-231), containing CTAR1 mutations (1-231-A5), or deleted for the entire cytoplasmic domain (1-187) were also tested with CYFP-TRAF2 and CYFP-TRAF3 for BiFC (Figure 2A). 1-231-NYFP fluorescence with CYFP-TRAF2 and CYFP-TRAF3 was lower than both LMP1-NYFP and A5-Y384G-NYFP combinations. Mutation of CTAR1 from 1-231 to 1-231-A5 reduced the fluorescence by about one half. The mutant containing only the transmembrane domain (1-187) had only minimal fluorescence. Western blotting for the truncated LMP1 mutants indicates that 1-231 is expressed at higher levels than full-length LMP1. Since LMP1 antibody does not recognize the transmembrane only mutant, blotting with the GFP polyclonal antibody that recognizes NYFP indicates that 1-187 is expressed at a lower level than full-length or 1-231 constructs. Because 1-187 was expressed at lower levels than the 1-231 constructs, it is unclear if reduced fluorescence was due to lower BiFC or impaired 1-187 expression. Greater expression of 1-231-NYFP compared to LMP1-NYFP did not result greater BiFC, indicating that greater expression alone does not induce greater BiFC. Decreased BiFC with 1-231-NYFP is likely due to steric hindrance of YFP domain association. The fact that fluorescence induced with the CYFP-TRAF2 and CYFP-TRAF3 with LMP1-NYFP and 1-231-NYFP was reduced by mutation or deletion of LMP1 signaling domains suggests that the BiFC of these combinations represents LMP1-signaling complexes. As with full-length LMP1-NYFP + CYFP-TRAF BiFC, 1-231-A5 that should have no TRAF binding still had greater fluorescence than 1-187-NYFP. It is possible that overexpression of BiFC plasmids in transient transfections may induce nonspecific BiFC.

To determine if C-terminally tagged TRAFs also induce BiFC, BiFC of LMP1-NYFP + TRAF2-CYFP and TRAF3-CYFP were performed (Figure 2A). BiFC was induced between LMP1-NYFP and TRAF-CYFP constructs (Figure 2A, gray bars). However, BiFC was not reduced by mutation of CTAR1 and CTAR2 with A5-Y384G compared to LMP1 (Figure 2A, gray bars). Similarly, LMP1 deletions lacking CTAR2 containing CTAR1 (1-231), containing CTAR1 mutations (1-231-A5), or deleted for the entire cytoplasmic domain (1-187) with TRAF2-CYFP or TRAF3-CYFP had low fluorescence that was not altered by mutation or deletion of CTAR1. Expression of TRAF2-CYFP and TRAF3-CYFP were confirmed (Figure 2B and data not shown, respectively). TRAF2-CYFP and TRAF3-CYFP contain a tandem triple-myc tag that increases their molecular weight. Since fluorescence was not reduced by CTAR1 and CTAR2 mutation or deletion, fluorescence resulting from these combinations does not likely represent LMP1-signaling complexes and may represent nonspecific binding.

**Figure 3:**
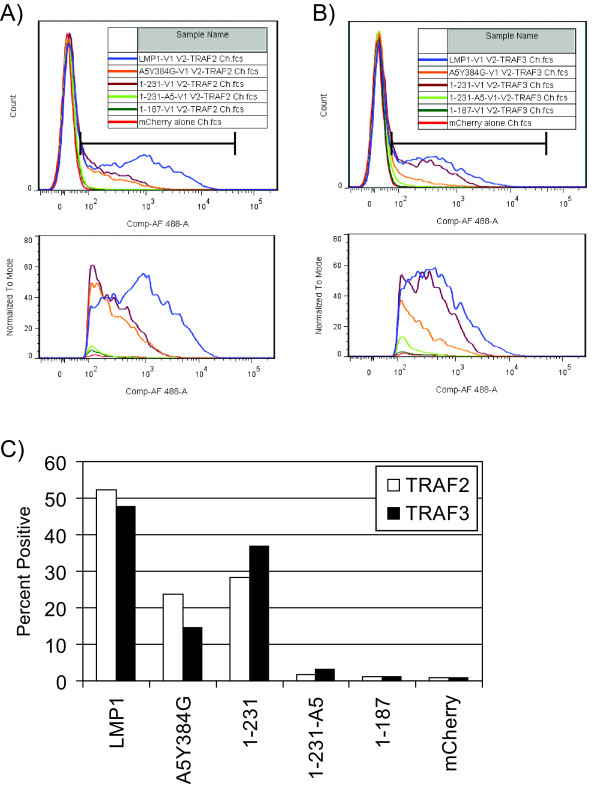
**BiFC Specificity with Decreased BiFC Construct Expression**. LMP1-NYFP and LMP1-NYFP mutants were transfected with CYFP-TRAF2 and CYFP-TRAF3 (Panel A and B, respectively) as in Figure 2 except that ten-fold less BiFC plasmids were used. Cells were analyzed for BiFC by flow cytometry as in Figure 2. Representative YFP histograms of 1 × 10^4 ^mCherry positive cells are depicted. The YFP positive cells were gated as indicated and expanded in the lower panels. Histograms for LMP1-NYFP (blue), A5-Y384G-NYFP (orange), 1-231-NYFP (purple), 1-231-A5-NYFP (light green), 1-187-NYFP (dark green), and mCherry alone (no BiFC plasmids, red) are displayed. Percent YFP positive cells in the mCherry positive population is displayed in panel C.

To determine if overexpression of BiFC proteins contribute to non-specific fluorescence, BiFC assays were performed with the same amount of mCherry tracer plasmid but ten-fold less BiFC plasmids. The YFP histograms of 1 × 10^4 ^mCherry positive cells from BiFC assays with lower BiFC plasmids are depicted in Figure 3A and 3B. As opposed to initial BiFC assays where greater than 90% of mCherry cells were also YFP positive (data not shown), approximately 50% or fewer of the mCherry positive cells were also YFP positive (Figure 3C). The YFP-positive population was gated as indicated and expanded in the lower panels (Figure 3A and 3B). CYFP-TRAF2 which should be able to bind both CTAR1 and CTAR2 induced strong fluorescence with LMP1-NYFP (Figure 3A, blue histogram) that was decreased by mutations in CTAR1 and CTAR2 (A5-Y384G) and deletion of CTAR2 (1-231) (Figure 3A, orange and purple histograms, respectively). 1-231-A5 and 1-187 had virtually no YFP positive cell (Figure 3A, green histograms) which was similar to cells transfected with mCherry alone (Figure 3A, red histogram).

Strong BiFC was also observed with CYFP-TRAF3 + LMP1-NYFP (Figure 3B, blue histogram). TRAF3 does not bind to CTAR2 and CTAR2-deleted 1-231-NYFP induces BiFC similar to LMP1-NYFP (Figure 3B, compare blue and purple histograms). A5-Y384G which is mutated for both signaling domains has decreased BiFC (Figure 3B, orange histogram) but still has fluorescence greater than the other mutants which should not bind TRAF3, 1-231-A5 and 1-187 (Figure 3B, green histograms). Transfection of less BiFC plasmids seems to recapitulate TRAF-LMP1 binding. TRAF2 can bind to both CTAR1 and CTAR2 and full-length LMP1-NYFP has greater fluorescence than 1-231-NYFP which is deleted for CTAR2 with CYFP-TRAF2. TRAF3 only binds to CTAR1 and LMP1-NYFP and 1-231-NYFP have similar fluorescence with CYFP-TRAF3. A5-Y384G which is predicted to bind neither TRAF2 nor TRAF3 still induces fluorescence greater than 1-231-A5 and 1-187 mutants (Figure 3C). Whether the residual BiFC with A5-Y384G is the result of specific or non-specific interaction is unclear.

**Figure 4:**
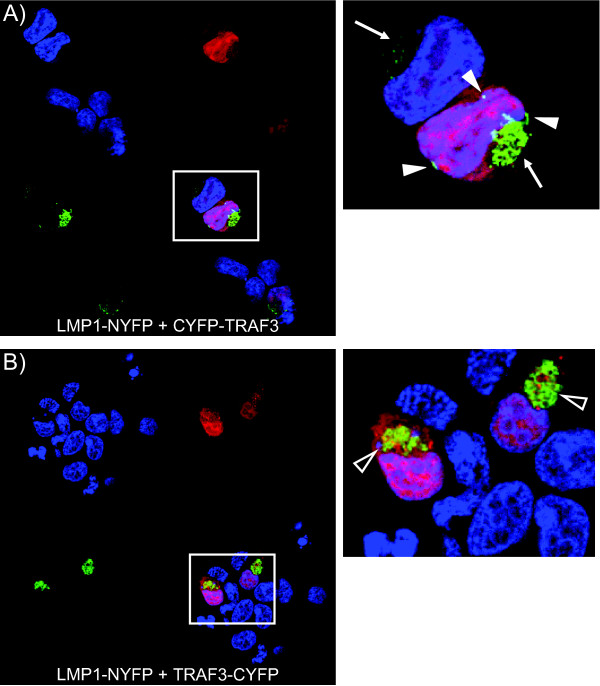
**Localization of LMP1 + TRAF BiFC**. Cells were plated on coverslips and transfected with the indicated BiFC plasmids. Twenty-four hours post-transfection cells were fixed, counterstained with DAPI, and mounted. High resolution images were acquired using a confocal microscope. Individual colors, blue (DAPI), red (mCherry transfection control), green (BiFC), and merged images are displayed. Representative cells (white boxes) were magnified and various fluorescence localizations are indicated: perinuclear (white arrows), membrane (white arrowheads), and cytoplasmic (open arrowheads).

As BiFC can be induced anywhere in the cell, cells can be observed for the localization of fluorescence by fluorescence microscopy. In contrast to fluorescence induced with NYFP-CTAR1/2 and CYFP-TRAF combinations, which was cytoplasmic, different combinations of full-length LMP1 and TRAFs fused at either the amino- or carboxyl-terminus to CYFP resulted in different patterns of staining (Figure 4). The two patterns correlated with the TRAF configuration and fluorescence. The TRAFs tagged at their amino-termini with the CYFP domain, which induced brigher fluorescence (Figure 2A, black and white bars), had fluorescence in two regions of the cell. As shown in the high magnification panel for LMP1-NYFP + CYFP-TRAF3 (Figure 4A), there was crescent-shaped bright fluorescence in a region that appeared to be perinuclear (Figure 4A, white arrows). Second, there were patches of fluorescence at the perimeter of the cell that are likely plasma membrane associated (Figure 4A, closed arrowheads). Both perinuclear and membrane fluorescence is consistent with previously described localization of LMP1-signaling complexes in LMP1-tranfected and EBV-infected cells [51,52]. The second fluorescence pattern, that was observed with the TRAFs tagged at the carboxyl terminus, which had lower MFI (Figure 2A, grey bars), was localized in discrete foci within cytoplasmic compartment, e.g. LMP1-NYFP + TRAF3-CYFP (Figure 4B, open arrowheads). These data correlate the LMP1-NYFP + CYFP-TRAF combinations with the greatest fluorescence, that were decreased by CTAR mutation or deletion, with previously described the membrane and perinuclear fluorescence of LMP1 signaling complexes.

### LMP1 + LMP1 BiFC

**Figure 5:**
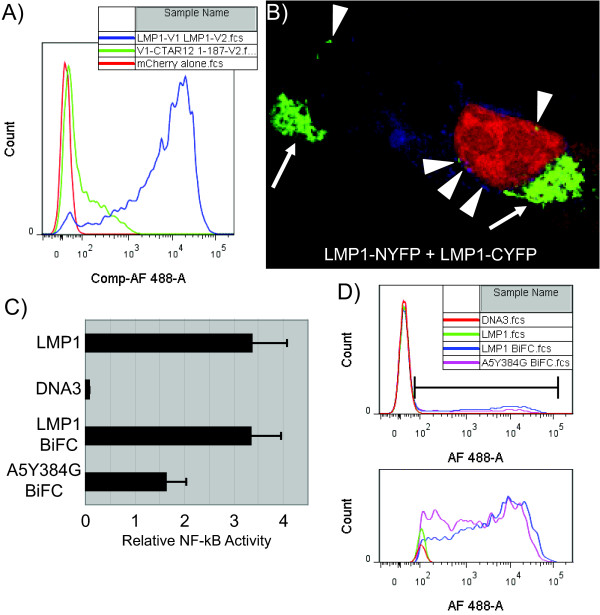
**LMP1 + LMP1 BiFC and NF-κB Activity**. Fluorescence complementation between the indicated LMP1 combinations was determined. BiFC was determined by flow cytometry as described above (Figure 3). Representative YFP histograms of 1 × 10^4 ^mCherry positive cells for LMP1-NYFP +LMP1-CYFP (blue), NYFP-CTAR1/2 + 1-187-CYFP (green), and mCherry alone (no BiFC plasmids, red) are displayed. Localization of LMP1-NYFP +LMP1-CYFP BiFC (panel B) was determined as in Figure 4. Perinuclear (white arrows) and membrane (white arrowheads) BiFC is indicated. Promoter reporter assays were performed by transfection of 293T cells with control pRL-SV40, pNF-κB-luc, and vector (DNA3), LMP1, LMP1-BiFC (LMP1-NYFP + LMP1-CYFP) or A5Y384G BiFC (A5-Y384G-NYFP + A5-Y384G-CYFP) plasmids. Forty hours post-transfection, cells were harvested and dual-luciferase assays were performed. Relative luciferase activity was determined by the firefly luciferase activity of the NF-κB reporter construct relative to the control Renilla luciferase activity. Each combination was performed in triplicate and the mean relative NF-κB activity is displayed. Error bars represent the standard deviations from the mean and the experiment has been repeated three times. In parallel BiFC was determined from the cells in the reporter assay (Panel D) as described above (Figure 3). Representative YFP histograms for DNA3 (red), LMP1 (green), LMP1 BiFC (blue), and A5Y384G BiFC (purple) are displayed.

The membrane domain of LMP1 is able to self-associate to induce signaling through the cytoplasmic domain of LMP1. To determine if LMP1 + LMP1 binding induces BiFC, assays were performed with LMP1 containing both YFP domains as partners (Figure 5). LMP1-NYFP + LMP1-CYFP induced strong fluorescence and NYFP-CTAR1/2 + 1-187-CYFP induced minimal fluorescence (Figure 5A, blue and green histograms, respectively). As with the LMP1-NYFP + CYFP-TRAF combinations, LMP1-LMP1 BiFC was localized to the perinuclear and plasma membranes of the cells (Figure 5B, white arrows and arrowheads, respectively). Switching the configuration of the YFP domains from the carboxyl to the amino terminus of LMP1 in different combinations resulted in lower levels of fluorescence complementation as measured by the mean fluorescence intensity by flow cytometry (data not shown). This suggests that LMP1-NYFP + LMP1-CYFP are the combination that most easily favors the assembly of YFP.

### Activation of NF-κB by BiFC Constructs

To assess the ability of LMP1 BiFC constructs to activate NF-κB, promoter reporter assays were performed with combinations of plasmids that induce BiFC. HEK-293T cells were cotransfected with an NF-κB promoter reporter (pNF-κB-Luc), transfection control Renilla luciferase (pRL-SV40), and effector plasmids. Effector plasmids include vector control (DNA3), N-terminally myc-tagged LMP1 (described previously [5], (LMP1)), LMP1-NYFP + LMP1-CYFP (LMP1 BiFC), and A5-Y384G-NYFP + A5-Y384G-CYFP (A5-Y384G BiFC). NF-κB-Luc promoter activity was plotted relative to the internal control Renilla luciferase activity (Figure 5C). As expected, LMP1 activates the NF-κB reporter compared to vector, approximately 45-fold. LMP1 BiFC plasmids activate the NF-κB reporter to a similar extent as LMP1. In parallel cells were analyzed for BiFC by flow cytometry (Figure 5D). LMP1 BiFC cells (blue histogram) also induced BiFC while vector and LMP1 cells had no YFP fluorescence as expected (red and green histograms, respectively). This indicates that LMP1 complexes which are inducing fluorescence are also inducing NF-κB signaling. A5-Y384G BiFC plasmids also induced the NF-κB reporter to about 50% of LMP1 and LMP1-BiFC NF-κB activation, approximately 22-fold above vector. This was unexpected as the A5-Y384G mutant was previously described to be defective in NF-κB activation and act as a dominant negative LMP1 [46]. A5-Y384G BiFC plasmids also induce fluorescence (Figure 5D, purple histogram), which is expected since LMP1-LMP1 binding is mediated by the transmembrane domain. The fact that the A5-Y384G BiFC plasmids induce activation of the NF-κB reporter suggests that the unanticipated A5-Y384G + TRAF BiFC (Figure 3A and 3B, orange histograms) is detecting association between the TRAFs and the A5-Y384G mutant that is inducing signaling. This also reinforces the use of BiFC in detecting physiological interactions.

### Transformation Assays with LMP1 BiFC Proteins

**Figure 6:**
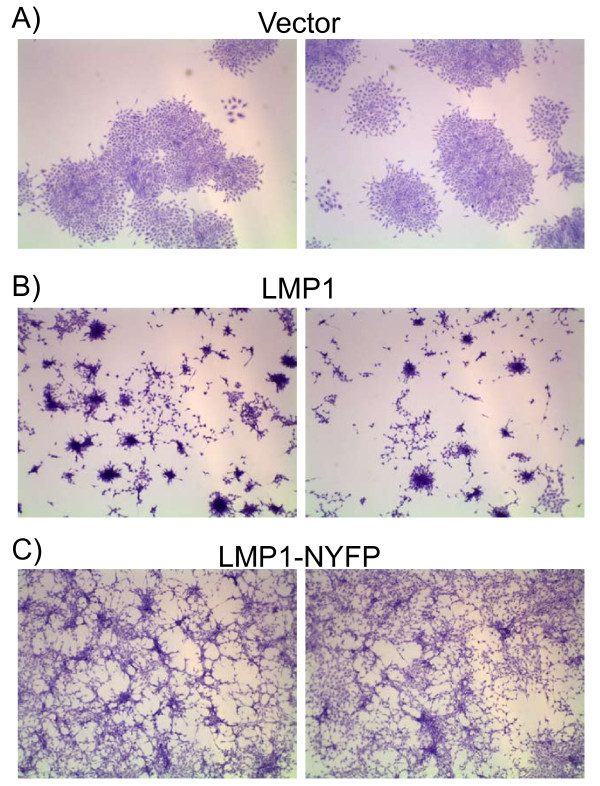
**Phenotype of LMP1-NYFP Stables**. LMP1-NYFP was subcloned into the pBabe packaging retrovirus. Control (Vector), LMP1, and LMP1-NYFP retrovirus were produced in HEK-293T cells. Rat-1 cells (rodent fibroblasts) were infected with the indicated retroviruses. Stable cell were selected and examined for phenotypic transformation. Subconfluent stable cells were fixed and stained with crystal violet. Representative micrographs taken with a dissecting microscope are presented.

**Figure 7:**
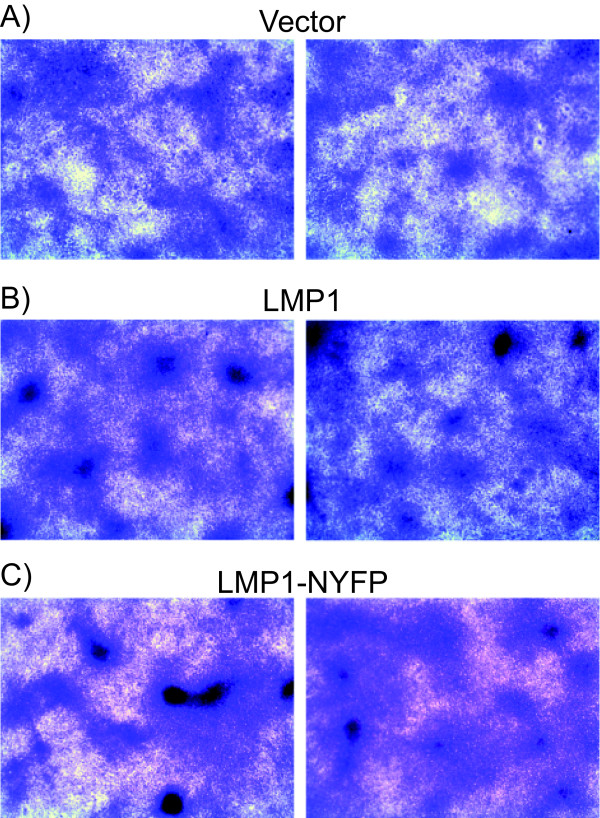
**Focus Formation with LMP1-NYFP**. Rat-1 monolayers were infected with Vector, LMP1, and LMP1-NYFP retrovirus and focus formation assays for contact-inhibition were performed. Ten days post-infection cells were fixed and stained with crystal violet for foci. Representative micrographs taken with a dissecting microscope are presented.

**Figure 8:**
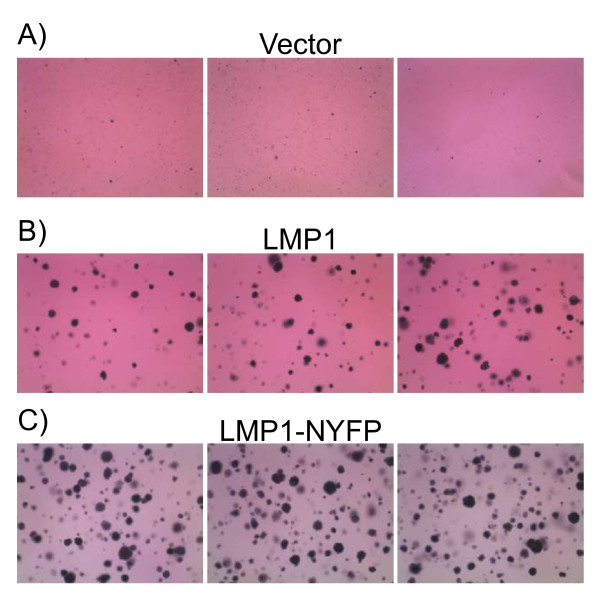
**Anchorage Independent Growth with LMP1-NYFP**. Rat-1 cells were infected with Vector, LMP1, and LMP1-NYFP retrovirus and stable cells were selected. Stable cells were analyzed for anchorage-independent growth in soft agar for 10 days and stained with MTT. Representative micrographs taken with a dissecting microscope are presented.

LMP1-TRAF and LMP1-LMP1 BiFC and LMP1-LMP1 NF-κB activation suggests that activation of NF-κB is not impaired by the NYFP and CYFP domains. Rodent fibroblast transformation requires both PI3K and ERK signaling through CTAR1 [6,7]. To determine if LMP1-NYFP is able to activate PI3K and ERK signaling, transformation assays were performed. LMP1-NYFP was subcloned into pBabe retrovirus expression vectors and used in transformation assays (Figures 6, 7, and 8). Rat-1 cells were infected with vector control, HA-tagged LMP1, and LMP1-NYFP retrovirus. Infected cells were selected with puromycin and examined after 5 days for altered growth properties (Figure 6). Vector control cells exhibited normal morphology and grew as a monolayer on tissue culture plates (Figure 6A), but both LMP1 and LMP1-NYFP expressing cells appeared spindly and grow on top of each other in patches (Figure 6B and 6C, respectively). In some areas the LMP1 cells grew in tight clumps that were not observed in LMP-NYFP cells, but LMP1-NYFP cells had an elongated morphology consistent with phenotypic transformation. To determine if LMP1-NYFP and 1-231-NYFP were able to induce transformation, Rat-1 cells were transduced with retrovirus and focus formation and soft agar assays were performed. In focus formation assays control cells were contact inhibited and stopped growing upon confluence (Figure 7A), while both LMP1 and LMP1-NYFP induced foci in Rat-1 cells (Figure 7B and 7C, respectively). Stably transduced cells were seeded into soft agar and observed for anchorage independent growth (Figure 8). Vector control cells did not grow in an anchorage independent manner (Figure 8A). Both LMP1 and LMP1-NYFP grew in an anchorage independent fashion and formed colonies in soft agar (Figure 8B and 8C, respectively. In parallel, stable cells were examined by western blotting for LMP1 expression (data not shown). In our previous studies LMP1 mutants containing amino acids 1-231 were sufficient to induce transformation [6,7] and 1-231-NYFP expressing retrovirus also induced focus formation in monolayers and colony formation in soft agar (data not shown). These data indicate that the presence of the YFP domain at the carboxyl-terminus of LMP1 does not impair LMP1 signaling through PI3K and ERK that are required for rodent fibroblast transformation.

## Discussion

The data presented in this study utilize the *in vivo *technique of BiFC to study assembly of LMP1 signaling complexes within cells. Fluorescence complementation was observed with LMP1 and TRAF2 or TRAF3. Mutation of CTAR1 and/or CTAR2 decreased fluorescence of LMP1 + TRAF combinations. LMP1 + LMP1 complementation was also observed. Both LMP1 + TRAF and LMP1 + LMP1 BiFC localized to perinuclear and membrane which is consistent with previously described LMP-signaling complexes. LMP1 mutants containing only the signaling domain of LMP1 induced cytoplasmic fluorescence with the TRAFs. LMP1 fusion proteins containing the YFP domain at the carboxyl-terminus of LMP1 induced NF-κB reporter activation and transformation of Rat-1 cells.

The data presented here reinforce the utility of using BiFC to study protein-protein interactions. However, several cautions are also highlighted by our studies. First, overexpression of proteins must be avoided. Transfection of ten-fold less plasmids resulted in diminished non-specific BiFC (Figure 3). Second, in the absence of structural information, different combinations and orientations of YFP domains on binding partners should be screened to find optimal BiFC partners to minimize steric hinderance. Third, correct cellular localization or mutations in known binding domains should be employed to ensure observed BiFC is physiologically relevant. BiFC between LMP1-NYFP and CYFP-TRAF2 or CYFP-TRAF3 was observed in physiological locations, perinuclear and membrane associated, and diminished by CTAR1 and CTAR2 mutation. In contrast, BiFC between LMP1-NYFP and TRAF2-CYFP or TRAF3-CYFP was observed in an unknown cytoplasmic compartment and was not diminished by CTAR1 and CTAR2 mutations. This indicates that TRAF-CYFP combinations will not yield insight into LMP1 binding and signaling. Last, it is important to ensure that the presence of the YFP domain does not affect critical properties of the protein. LMP1 BiFC proteins were able to activate NF-κB and induce rodent fibroblast transformation.

LMP1-binding proteins were initially identified using Y2H screens with the cytoplasmic domain of LMP1 [48]. Although Y2H screens are powerful tools for identifying and characterizing protein-protein interactions, Y2H requires interacting proteins to be transported to the nucleus to induce transcription of reporter genes which generally precludes the inclusion of transmembrane domains. LMP1 signaling occurs in the cholesterol rich lipid raft domains of the membrane [20-26]. The contribution of the membrane domain of LMP1 to recruitment of downstream effector proteins can generally not be determined by Y2H. In contrast, bimolecular fluorescence complementation does not require nuclear localization and can be performed within mammalian cells. Previous immunofluorescence for LMP1 or tagging of LMP1 with green or red fluorescent proteins resulted in fluorescence in membrane patches as well as fluorescence in perinuclear regions of the cell (e.g. [51,52]). BiFC with both LMP1 + TRAF and LMP1 + LMP1 combinations in the current study induced membrane and perinuclear fluorescence as well. This suggests that the fluorescence resulting from BiFC is induced by LMP1 signaling complexes within a physiological context and demonstrates the utility of BiFC to study the assembly of LMP1-signaling complexes in membrane of mammalian cells.

The CTAR2 signaling domain has been defined as the terminal three amino acids YYD of LMP1. There was concern that addition of the YFP domain to C-terminus might inhibit CTAR2 signaling. However, several of our experiments suggest that this is not the case. First, deletion of CTAR2, LMP1-NYFP to 1-231-NYFP, resulted in a decrease in BiFC with CYFP-TRAF2 which can bind to either CTAR1 or CTAR2. Second, the majority of the NF-κB activation is a result of CTAR2 [16] and LMP1 BiFC plasmids were as effective as LMP1 in induction of the NF-κB reporter. Our studies suggest that the presence of the NYFP domain functions as a suppressor for the Y384G mutation or act as a gain of function for CTAR2 signaling. Although we were concerned that CYFP-TRAF2 binding to A5-Y384G-NYFP was an artifact. The NF-κB reporter activation suggests that TRAF2 is binding to A5-Y384G-NYFP to induce signaling. The fusion protein junction may create a new or secondary CTAR2 sequence. Mutation of CTAR2 from wild-type (YYD) to GYD at the C-terminus of LMP1 abrogates CTAR2 signaling. A5-Y384G-NYFP creates the sequence GYDIDGGGGSGGGGS at the junction between LMP1 and NYFP, where the GYD is the mutated CTAR2, ID is contributed by a restriction enzyme site, and GGGGSGGGGS is the linker sequence of the BiFC vector. Our hypothesis is that Y385 and D388 in the junction sequence are able to substitute for Y384 and D386 in the wild-type CTAR2. In any case, this new TRAF2 binding to A5-Y384G-NYFP was detected by BiFC and its function was confirmed by the NF-κB reporter activation.

Several recently developed techniques have been developed to not only characterize known protein-protein interactions but screen for new interacting partners via BiFC [53-56]. LMP1-NYFP could be used as a bait protein to screen an expression library cloned downstream of CYFP. Fluorescent cells can be identified and isolated using fluorescence activated cell sorting and the interacting genes can be identified by reverse genetics. Performing such a screen in mammalian cells could have several advantages over a technique like Y2H. Since these screens are performed in mammalian cells, all physiologically relevant accessory proteins would also be present. Because of the flexible linker region, it may also be possible to identify indirect targets of LMP-mediated signaling, i.e. downstream proteins recruited to LMP1 by the TRAFs which might not directly bind LMP1. As described above, our BiFC data suggest that TRAF2 binding to CTAR2, which is an indirect binding, is intact. As the association of the YFP domains in BiFC is considered to be nearly irreversible, it may be possible to capture low-affinity or transient interactions as well [44]. BiFC may be valuable tool to characterize the proteins recruited to LMP1 within the context of the dynamic signaling environment.

Fluorescence based assays are powerful tools to examine protein-protein interactions. Since LMP1 + TRAF and LMP1 + LMP1 association were able to be visualized by BiFC, it suggests that the LMP1 signaling complex could also be probed using a more rigorous fluorescence techniques like fluorescence resonance energy transfer (FRET). FRET requires that donor and acceptor proteins are within 10 nm of each other. BiFC combinations that result in high fluorescence represent likely combinations for positions for FRET partners. Dynamic interactions could be rigorously examined using an approach such as FRET. Recently a technique that uses a combination of BiFC and FRET to study the assembly of ternary complex of transcription factors in real-time within the nucleus of the cells has been described [57-59]. Such approaches could be applied to the assembly of signaling complexes. As new proteins recruited to the LMP1-signaling complex are identified, the assembly of the LMP1-signaling complex in real-time in the membrane of cells could be determined. Furthermore, fluorescence-based binding assays could be used to screen compound libraries for inhibitors of LMP1 signaling.

## Conclusions

Together these data indicate that BiFC serves as a novel in vivo platform to study LMP1 signaling in the membrane of mammalian cells, including NF-κB, PI3K and ERK signaling.

## List of Abbreviations

EBV: Epstein-Barr virus; LMP1: latent membrane protein 1; BiFC: bimolecular fluorescence complementation; TRAF: tumor necrosis factor receptor associated factor; NF-κB: nuclear factor-κB; CTAR: C-terminal activating region; CTAR1/2: LMP1 mutant containing only the cytoplasmic domain; A5: LMP1 CTAR1 mutant; Y384G: LMP1 CTAR2 mutant; A5-Y384G: LMP1 CTAR1 and CTAR2 mutant; PI3K: phosphoinositide 3-kinase; ERK: extracellular-signal-regulated kinase; YFP: yellow fluorescent protein; NYFP: N-terminal YFP fragment; CYFP: C-terminal YFP fragment; Zip: leucine zipper domain; RING: really interesting new gene; HEK: human embryonic kidney; SDS: Sodium dodecyl sulfate; DAPI: 4'-6-Diamidino-2-phenylindole; PBS: phosphate buffered saline; MFI: mean fluorescent intensity; MTT: methylthiazolyldiphenyl-tetrazolium bromide; Y2H: yeast two-hybrid; kDa: kilodaltons; FRET: fluorescence resonance energy transfer; RFUMS: Rosalind Franklin University of Medicine and Science

## Competing interests

The authors declare that they have no competing interests.

## Authors' contributions

PT cloned BiFC vectors and performed BiFC assays. AE participated in data acquisition and analysis. DE conceived of the study and its design. DE also was responsible for overseeing data acquisition, analysis, and drafted the manuscript. All authors read and approved of the final manuscript.
